# Update of Clown Nose-Like Lesion, a Underrecognized Manifestation of Metastatic Malignancies and Genetic Cancer Predisposition Syndromes

**DOI:** 10.3389/fmed.2021.673336

**Published:** 2021-05-13

**Authors:** Bei Zhao, Ling Chen, Jinfeng Liao, Zhen Xie, Xia Lei, Zhu Shen

**Affiliations:** ^1^Department of Dermatology, Institute of Dermatology and Venereology, Sichuan Academy of Medical Sciences & Sichuan Provincial People's Hospital, Chengdu, China; ^2^Department of Dermatology, Daping Hospital, Army Medical University, Chongqing, China; ^3^School of Medicine, University of Electronic Science and Technology of China, Chengdu, China

**Keywords:** skin metastases, lung cancer, genetic syndromes, manifestation, nose, clown nose-like lesion

## Abstract

Clown nose-like lesion refers to the manifestation of a reddish or skin-colored bulge on the tip of the nose or the manifestation of bulbous tip of the nose. More and more clinical cases show that clown nose-like lesion can also be the indication of some genetic syndromes, not just the manifestation of metastatic visceral tumor as it initially proposed. However, the clinical features of clown nose-like lesion indicated by metastatic malignancies, genetic cancer predisposition syndromes or primary diseases involving the nasal tip are lacking. In this study, patients with clown nose-like lesion in our clinical practices and from published literatures were collected and reviewed. We found that clown nose-like lesions caused by metastatic malignancies including lung cancer are often solitary and more common in male (24/31) older individuals (average age 62.3, ranging 40–78 years old). In addition, they usually appear for a short time, and are prone to be misdiagnosed as primary nasal diseases, leading to a poor prognosis (all patients with data available died within 4 months). Clown nose-like lesions associated with genetic cancer predisposition syndromes usually develop at a young age (mean age 15.3) with female preference (9/10). They are accompanied by multiple-systemic involvements, including low hair volume, developmental delay, cancer predisposition or neurological diseases. They show slow development and often positive family history (6/10). These two kinds of clown nose-like lesions are often asymptomatic, which delays the diagnosis and treatment of underlying malignancies or syndromes. In brief, the term of clown nose-like lesion is underrecognized, and should be updated. Clown nose-like lesions can serve as indicators to at least three categories of clinical issues: metastatic visceral tumors, genetic syndromes, and primary diseases involving the nasal tip. Increased awareness of clinical features of updated clown nose-like lesions can alert physicians to these underlying malignancies or syndromes, render earlier detection of associated medical issues, and allow for genetic counseling of family members.

## Introduction

Clown nose-like lesion refers to the manifestation of a reddish or skin-colored bulge on the tip of the nose or the manifestation of bulbous tip of the nose, resembling the fake nose of a clown (1). Initially, clown nose-like lesion was considered as a indicator of cutaneous metastatic malignancies, including lung cancer (2), renal clear cell carcinoma (3), rhabdomyosarcoma (4), cervical cancer (5), and liver cancer (6). In clinical practice, however, it can also be a cue for genetic syndromes, such as Tricho-Rhino-Phalangeal syndrome (7), as well as some primary nasal diseases including infections such as leishmaniasis (8), inflammations such as rosacea (9), and tumors such as keratoacanthoma (10).

In this study, patients with clown nose-like lesions in our clinical practices and from published literatures were collected and reviewed. They were divided into three categories: metastatic visceral tumors, genetic syndromes, and primary diseases involving the nasal tip. Detailed clinical features on the gender, age, skin lesion characteristics, nasal skin lesion symptoms, initial diagnosis, and outcome of these cases were analyzed. Increased awareness of these clinical features of updated clown nose-like lesions can alert physicians to the underlying malignancies or genetic syndromes, and render earlier detection of associated medical comorbidities.

## Methods

### Patients

We collected clinical cases with clown nose-like lesions who were admitted to our dermatology departments. Their initial diagnoses were made by a dermatologist at admission, and confirmed/corrected by three independent dermatologists with the help of histopathology and/or medical imaging. This clinical case survey was approved by Medical Ethics Committee in Sichuan Academy of Medical Sciences & Sichuan Provincial People's Hospital.

### Literature Review

Published cases with clown nose-like lesions were retrieved from the databases of PubMed and Google Scholar. Medical Subject Heading (MeSH) included “nose” or “nasal” combined with “syndrome,” “genetic,” “cancer,” “tumor,” or “carcinoma.” Only articles published in English were included.

### Data Analysis

These cases were divided into three categories: metastatic visceral tumors, genetic syndromes, and primary diseases involving the nasal tip. Their medical records were collected and reviewed. Detailed clinical features on the gender, age, skin lesion characteristics, nasal skin lesion symptoms, initial diagnosis, and outcome of these cases was analyzed.

## Results

### Demographic and Clinical Characteristics

**Figure 1:**
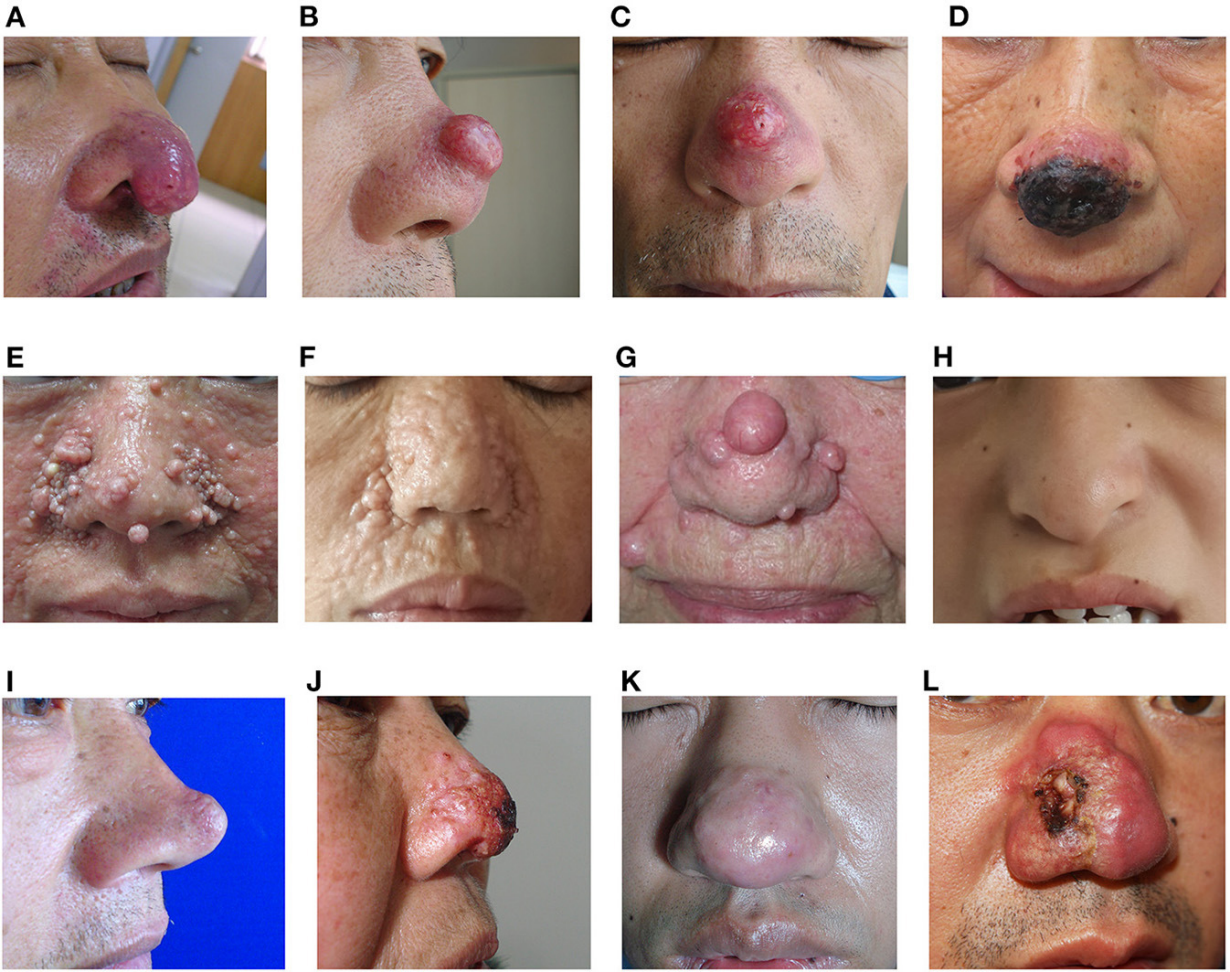
Clinical images of clown nose-like lesions. **(A–D)** Clown nose-like lesions secondary to metastatic visceral tumors. **(A)** A 65-year-old man with lung squamous cell carcinoma (case 4 in Table 1), initially misdiagnosed as rosacea; **(B,C)** a 65-year-old man with lung squamous cell carcinoma (case 2 in Table 1), initially misdiagnosed as folliculitis; **(D)** a 63-year-old woman with lung squamous cell carcinoma (case 1 in Table 1), initially misdiagnosed as basal cell carcinoma. **(E–H)** Clown nose-like lesions nose as manifestations of genetic syndromes. **(E)** A 34-year-old woman with tuberous sclerosis (case 6 in Table 3); **(F)** a 40-year-old woman with multiple familial trichoepithelioma (case 5 in Table 3); **(G)** a 85-year-old woman with Brooke-Spiegler syndrome (11); **(H)** a 13-year-old girl with Tricho-Rhino-Phalangeal Syndrome in our department. **(I–L)** Primary skin disease of clown nose-like lesions. **(I)** A 47-year-old man with sebaceous gland hyperplasia (case 12 in Table 4); **(J)** a 62-year-old woman with superficial skin mycosis (case 8 in Table 4); **(K)** a 42-year-old man with rosacea (case 10 in Table 4); **(L)** a 34-year-old man with nasal squamous cell carcinoma (case 9 in Table 4).

In total, 31 cases with clown nose-like lesions from metastatic visceral tumors and 10 cases with clown nose-like lesions from genetic syndromes, as well as 20 kinds of primary diseases involving the nasal tip, were collected (Figure 1). Their detailed demographic information and clinical characteristics were displayed in Tables 1–4.

**Table 1:** Nasal tip cutaneous metastasis from lung carcinoma.

**Sex/Age**	**Description of nasal lesion**	**Course of clown nose-like lesion**	**Initial diagnosis**	**Lung histopathology**	**Prognosis**	**References**
F/76	A blue-black spherical mass	N/A	Haemangioma	Oat cell carcinoma	Died in 3 M	(12)
M/67	A subcutaneous lump	4 M	Rhinophyma	Squamous cell carcinoma	N/A	(12)
M/71	N/A	N/A	Rosacea	Squamous cell carcinoma	Died immediately	(13)
M/65	Enlarging nasal tip	4 W	Cellulitis	Adenosquamous cell carcinoma	Died in several weeks	(14)
M/58	Nodules	1 M	Metastatic tumor	Not described	Died in 3 M	(15)
M/63	N/A	N/A	Tumor	Anaplastic large cell carcinoma	Died in 2 W	(16)
M/59	Nodules	6 W	Mass	Squamous cell carcinoma	N/A	(17)
M/64	A cutaneous tumor	3 M	Furuncle	Large cell undifferentiated carcinoma	Died in 6 M	(18)
M/74	Fuchsia nodules	N/A	Folliculitis	Squamous cell carcinoma	Died in 3 M	(19)
M/62	A round erythematous nodule	5 W	Tumor	Squamous cell carcinoma	Died in 5 M	(2)
M/76	A round erythematous tumor	4 M	Rhinophyma	Squamous cell carcinoma	Died in 1 M	(2)
M/57	Tender, oval and erythematous nodules	1 M	Rhinophyma	Squamous cell carcinoma	Died in 9 M	(2)
F/63	Pale pink patches and black scabs	1 M	Basal cell carcinoma	Squamous cell carcinoma	Died in 6 M	Our case 1
M/65	Red nodules with clear border	5 W	Folliculitis	Squamous cell carcinoma	Died in 7 M	Our case 2
M/70	Cauliflower-like mass	2 M	Metastatic tumor	Squamous cell carcinoma	Died in 2 M	Our case 3
M/65	Red nodule	<1 M	Rosacea	Squamous cell carcinoma	N/A	Our case 4

*“F” and “M,” female and male in Sex column; “Y,” “M,” and “W,” year, month, and week in course of clownlike nose column; “N/A,” data not available*.

### Clown Nose-Like Lesions Secondary to Metastatic Visceral Tumors

#### Nasal Tip Cutaneous Metastasis From Lung Carcinoma

Internal malignant tumors can metastasize to other organs including the skin (49, 50). When looking up the frequency of metastasis of lung carcinomas, there are some preferential sites, as the bone 34.3%, the brain 28.4%, the adrenals 16.7%, and the liver 13.4%, depending on the availability of research data (51). Lung carcinoma metastasis to the skin, especially the nose, is rare and easily overlooked.

In this study, a total of 16 cases of lung cancer metastasis with clown nose-like lesions were collected (Table 1). We found they usually presented as active or fixed, hard or soft, single or multiple, painless nodules. They had a variety of colors, ranging from flesh-colored to red-purple, blue-black, and they ranged in diameter from 5 mm to 6 cm (Figure 1). The average age of the 16 cases was 65.9 (ranging 57–76 years old), indicating that lung cancer and its nasal metastasis have a higher incidence in older individuals. Only two of the 16 cases were women. Considering that the incidence of lung cancer is not much different between men and women (52), it further indicated that nasal metastasis may be more likely to occur in male (14/16) older lung cancer patients (average age 65.4). In addition, most lung cancer patients (11/16) with clown nose-like lesions have been demonstrated to be metastatic squamous cell carcinoma by histopathology. Moreover, the nose is the most noticeable area of the face, and patient with nasal involvement are more likely to present to a dermatology clinic, while ignoring the signs and symptoms that accompany systemic diseases, especially when there is no history of internal malignancies. In this study, we showed that clown nose-like lesions usually appear for a short time (<2 months), and most cases (14/16) were misdiagnosed as primary nasal skin diseases, especially rosacea or furuncle, at the first visit. Finally, the prognosis of lung cancer with skin metastasis is poor. Skin metastases are usually accompanied by internal metastases in other organs, such as brain, liver, bone and adrenal glands (our *Case 3*). It has been reported that the average survival time after metastasis was 5 months (53). In this retrospective analysis, patients died about 4 months after the diagnosis of clown nose-like lesion metastasis, suggesting poorer prognosis (Table 1).

#### Nasal Tip Cutaneous Metastasis From Other Carcinomas

In addition to the 16 cases of lung cancer metastasis mentioned above, we analyzed 15 cases suffering from nasal metastasis secondary to renal clear cell carcinoma, rhabdomyosarcoma, cervical cancer, chordoma, liver cancer, acute myeloid leukemia, basal laryngeal squamous cell carcinoma, squamous cell carcinoma of the larynx, thyroid cancer, hypopharyngeal cancer, hypopharyngeal squamous cell carcinoma, esophageal squamous cell carcinoma and esophageal cancer, respectively (Table 2). In these cases, the course of clown nose-like lesion lasted from several weeks to 1 year (mean 3 months), and nasal manifestations usually appeared as swelling, purplish red bumps or nodules. Similar to lung cancer metastasis, patients with clown nose-like lesions secondary to these cancers were usually older than 50 (average age 55.1, ranging 6–78 years old), especially male (10/15) older patients (average age 58, ranging 40–78 years old). When we put all the tumors together (Tables 1, 2), we can still find gender preference of clown nose-like lesion in male elderly (24/31, average age 62.3, ranging 40–78 years old).

**Table 2:** Nasal tip cutaneous metastasis from other carcinomas.

**Sex/Age**	**Primary tumor**	**Nasal lesions**	**Course of clownlike nose**	**Chief complaint organ at first visit**	**Treatment**		**Prognosis**	**References**
					**Nasal tip**	**System therapy**		
F/55	Renal clear cell carcinoma	Vascular erythema, papules	1 Y	Kidney	N/A	Local radiotherapy	N/A	(3)
F/6	Rhabdomyosarcoma of nasal cavity	Nose lumps	6 M	Nasal tip	resection	Partial resection	N/A	(4)
F/48	Cervical cancer	Purple-red and swollen; dilated capillaries	3 M	Nasal tip	Local radiotherapy	Resection	Purplish redness faded after 5 years	(5)
F/64	Cervical cancer	Swelling	3 M	Cervix	Radiotherapy	Surgery plus radiotherapy	Died after 3 M	(20)
M/40	Sacrococcygeal chordoma	Small hard nodules	2 M	Spine	N/A	N/A	N/A	(21)
M/78	Liver cancer	Violet bump	N/A	N/A	N/A	N/A	Died after 20 M	(6)
M/18	Acute myeloid leukemia	Violet nodules, bumps	1 M	N/A	N/A	Chemotherapy	Getting well gradually	(22)
M/72	Basal laryngeal squamous cell carcinoma	Tumors prone to bleeding	N/A	Throat	N/A	Surgery, radiotherapy and chemotherapy	Died after 2 M	(23)
M/77	Squamous cell carcinoma of the larynx	Nose lumps	3 M	Throat	Partial resection	Radiotherapy or chemotherapy	N/A	(24)
M/54	Thyroid cancer	N/A	2 M	Neck	N/A	Chemotherapy	Died after 2 M	(25)
M/74	Hypopharyngeal cancer	Red nodules	2 W	Hypopharynx, esophagus	N/A	Chemotherapy, surgery and radiotherapy	N/A	(26)
M/54	Hypopharyngeal squamous cell carcinoma	Dilation of nasal capillaries, central ulcer	2 M	Hypopharynx	N/A	Chemotherapy	N/A	(27)
F/74	Esophageal squamous cell carcinoma	Painless violet-like red nodules	6 W	Esophagus	N/A	N/A	N/A	(28)
M/54	Esophageal squamous cell carcinoma	Dilated nasal blood vessels and central necrosis	N/A	Esophagus	Surgery	Surgery, radiotherapy	N/A	(29)
M/59	Esophageal cancer	Hemispherical mass with clear boundaries	N/A	Nasal tip	N/A	Chemotherapy	Died after 18 M	(30)

*“F” and “M,” female and male in Sex column; “Y,” “M,” and “W,” year, month, and week in course of clownlike nose column; “N/A,” data not available*.

The primary tumors were diagnosed first in most cases (12/15 cases), however, in some cases like the nasal tip metastasis from rhabdomyosarcoma, cervical cancer, and esophageal cancer in Table 2, the diagnosis of clown nose-like lesion even preceded the primary tumors. Consistent with those in lung cancers, clown nose-like lesions in these tumors also indicate poor prognosis, and patients died about 9 months after the diagnosis of clown nose-like lesion metastasis.

### Clown Nose-Like Lesion as a Manifestation of Genetic Syndromes

Nasal lesions can also be as a manifestation of some genetic syndromes. We collected or referred to 10 cases of genetic syndromes with clown nose-like lesions, including Hernandez syndrome, Tricho-Rhino-Phalangeal Syndrome type I, multiple familial trichoepithelioma, tuberous sclerosis, and Brooke-Spiegler Syndrome (Table 3 and Figure 1). All cases developed nasal manifestations at a young age (mean 15.3 years old). Nine of them were women, suggesting a possible female predilection. Their nasal lesions usually presented as multiple hypertrophic nodules or papules, except for bulbous tip of the nose in patients of Tricho-Rhino-Phalangeal Syndrome type I. In addition, clown nose-like lesions were always accompanied by other obvious characteristics, such as cancer predisposition, sparse hair, intellectual problems, and/or neurological diseases (e.g., epilepsy).

**Table 3:** Clown nose-like lesion as a manifestation of genetic syndromes.

**Sex/Age**	**Diagnosis**	**Characteristics of nasal lesions**	**Time of skin lesion**	**Involvement other than nose**	**Family history**	**References**
F/16	Hernandez syndrome	A large bulbous nose with thickened alae nasi and septum	Since age 8	Convulsive disorder, psychomotor retardation, and obesity	N/A	(31)
F/4	Multiple familial trichoepithelioma	Multiple round smooth translucent solid nodules and papules	Since age 2	N/A	Grandmother and mother	(32)
F/40	Multiple familial trichoepithelioma	Numerous flesh-colored round solid nodules and papules	Since age 10	Trichoblastic carcinoma	Mother	Our case 5
F/8	Tricho-Rhino-Phalangeal Syndrome-I	Round nose	Since age 8	Short and sparse hair, sparse outer eyebrows, short stature, thin nails, and curved fingers	Mother and grandmother	(33)
M/4	Tricho-Rhino-Phalangeal Syndrome-I	Thick and pear-shaped nose	Since age 4	Thinning hair, thinning of the outer eyebrows, thin upper lip, premature fusion of the epiphyses, difficulty in eating since childhood	N/A	(7)
F/1.4	Tricho-Rhino-Phalangeal Syndrome-I	Spherical nasal tip	After birth	Growth retardation after birth, sparse hair, thin upper lip, prominent ears and forehead, small chin, short hands, and feet	Grandfather, uncle and mother	(34)
F/34	Tuberous sclerosis	Light red rice grains to pea-sized hard pimples	Since age 30	Mental retardation, epilepsy, hemoptysis, multiple subungual fibromas, multiple hamartomas of the liver and kidney, pulmonary lymphangiomyomatosis	Mother and son	Our case 6
F/15	Tuberous sclerosis	Pale red verrucous papules	Since age 10	Mental retardation, epilepsy, multiple fibromas. Brain MRI and CT showed several cortical and subcortical nodules, subependymal nodules	N/A	(35)
F/26	Tuberous sclerosis	Pale red pimples	Since age 26	Epilepsy, brain imaging showed multiple cortical nodules and cortical malformations, but no subependymal nodules. Torso CT showed bilateral lumbosacral joint sclerosis	Two sisters have epilepsy	(36)
F/85	Brooke-Spiegler syndrome	Multiple papulonodular lesions, increasing in number over the years	Since age 55	Metastatic cylindrocarcinoma	N/A	(11)

*“F” and “M,” female and male in Sex column; “Y,” “M,” and “W,” year, month, and week in course of clownlike nose column; “N/A,” data not available*.

### Primary Diseases That Can Present With Clown Nose-Like Lesions

Primary diseases with clown nose-like lesions may be related to infections, inflammations, tumors, and other associated diseases (Table 4 and Figure 1). These diseases may present with nasal manifestations such as erythema, papules or nodules, and can be diagnosed through clinical features or laboratory tests. Clown nose-like lesions caused by infections or inflammations are relatively easy to diagnose via pathogen or clinic identifications. Neoplasm-associated clown nose-like lesions were common among middle-aged and elderly patients. However, for neoplasm-associated clown nose-like lesions, it was difficult to judge whether the tumor was benign or malignant, nor primary or secondary only by nasal manifestations. In this case, tissue biopsy and a thorough history including a review of systems were necessary to help make the final clinical diagnosis.

**Table 4:** Primary diseases that can present with clown nose-like lesions.

**Classification**	**Diseases**	**Nasal symptoms**	**Diagnosis**	**References**
Infection	Cutaneous Leishmaniasis	Erythema, verrucous hyperplasia	Identification of Leishmania by smear or culture	(37)
	Blastomycosis	Verrucous nodules	Histopathology	(38)
	Deep skin mycosis	Erythema, papules, scabs	Microscopic examination or culture of fungi	Our case 7
	Superficial skin mycosis	Erythema, papules, scaling	Microscopic examination or culture of fungi	Our case 8
Tumor-associated	Basal cell carcinoma	Erythema, plaque, ulcer	Histopathology	(39)
	Squamous cell carcinoma	Plaque, verrucous hyperplasia, central ulcer	Histopathology	Our case 9
	Keratoacanthoma	Papules, nodules	Histopathology	(10)
	Sebaceous carcinoma	Light red nodules	Histopathology	(40)
	Trichoblastoma	Nodules	Histopathology	(41)
	Microcystic adnexal carcinoma	Yellow or red nodules	Histopathology	(42)
	Cutaneous T-cell lymphoma	Scabs, bloody discharge	Histopathology	(43)
	Rhabdomyosarcoma	Smooth surface, red hard patches	Histopathology	(44)
	Folliculosebaceous cystic hamartoma	Tawny skin mass	Histopathology	(45)
	Cutaneous sinus histiocytosis	Papules, nodules	Histopathology	(46)
Inflammation	Rosacea	Erythema, nodules, and nasal glands	Clinic	Our case 10
	Acne	Nodules, cysts	Clinic	Our case 11
Others	Sebaceous gland hyperplasia	Light-red or light-yellow nodular hyperplasia	Histopathology	Our case 12
	Pseudolymphoma	Red smooth hard nodules	Histopathology	(26)
	Sinonasal sarcoidosis	Papules, nodules	Histopathology	(47)
	Juvenile xanthogranuloma	Yellow-red nodules	Histopathology	(48)

*“N/A,” data not available*.

## Discussion

The clown nose-like lesion is a term that refers to the manifestation of a reddish or skin-colored bulge on the tip of the nose or the manifestation of bulbous tip of the nose (1). Although clown nose-like lesion had been classically considered an indicator of cutaneous metastatic malignancies (e.g., lung cancer), there can be various other causes. Therefore, clinical issues behind clown nose-like lesions need to be considered comprehensively. In this study, patients with clown nose-like lesions in our clinical practices and from published literatures were collected. They were divided into three categories and reviewed: metastatic visceral tumors, genetic syndromes, and primary diseases involving the nasal tip.

For primary diseases with clown nose-like lesions, we classify them by infectious diseases, neoplasm-related diseases, inflammatory diseases, and other diseases. Infectious diseases have no obvious age tendency. The patient's living area or occupation should be noted whether it is possible to come into contact with animals or parasites. It's often necessary to rely on pathogenic examination or histopathological examination to help the diagnosis. Primary neoplastic diseases are usually found in patients with more than 50 years old. Ulcers may also present alongside the nasal nodules. The type of tumor needs to be confirmed by histopathological examination. Inflammatory diseases, such as rosacea, are common diseases. People may mistake rosacea for acne, eczema or even the allergic skin reaction. Rosacea presents main symptoms including facial flushing, irritated skin, and pimples as well as other symptoms like blushing easily. It can often be clinically identified or with the help other instruments (e.g., dermatoscopy).

In some cases, clown nose-like lesions can serve as early indicators to genetic syndromes, including Hernandez syndrome and Tricho-Rhino-Phalangeal syndrome (31–33). This is of great clinical significance, because genetic syndromes with skin lesions and multisystemic involvement, including cancer predisposition, are often underrecognized. The majority of skin lesions are asymptomatic, easily leading to delayed diagnosis of underlying cancers in dermatological practice. We take Tricho-Rhino-Phalangeal syndrome as an example. It has three types, and its common manifestations include enlarged round nose, sparse hair, bone, and joint abnormalities (54). Unlike clown nose-like lesion caused by other causes, the nose in Tricho-Rhino-Phalangeal syndrome is usually enlarged into pear-shaped bulb and asymptomatic. More importantly, patients are often accompanied by impairments of other systems such as intellectual, musculoskeletal, and neurological systems. Dermatologists should have a systematic approach and recognize the characteristic clown nose-like lesion early, and render earlier genetic screening of patients and their family members.

Clown nose-like lesion was initially considered as a indicator of cutaneous metastatic malignancies, including lung carcinomas. Since most visceral malignant tumors have a poor prognosis, early diagnosis is very important. For older patients (usually more than 50 years old), careful consideration should be given to decide whether the asymptomatic clown nose-like lesion is a metastatic malignancy, especially when it appears for only a short period of time (within a few weeks). Detailed medical history, tissue biopsy, and systematic examination are necessary. The percentage of patients with cutaneous metastases originating from visceral cancers are ~0.7–9% (55), in which lung cancer metastasis happened in 1–12% of cases (2, 56). Metastatic transmission to the skin can occur in the following ways: lymphatic transmission, blood-borne transmission, direct continuity, and rarely iatrogenic implantation (57). Under most circumstances, the tumor will preferentially metastasize to the nearby skin, and distant metastasis to the skin is relatively rare (2). The mechanism of cancer metastasis to the nose has not been fully elucidated. As reported previously (58), tumor cells can be transported to the nose through the pulmonary blood vessels, lymphatic circulation, and vertebral venous plexus without any valves. In addition, an emboli traveling through the arteries was also a possible route of metastasis (59). Tumor emboli may enter the pulmonary vein, then the systemic circulation through the left atrium, and enter the cavernous body into the blood supply site. Another possible mechanism of nasal tip metastasis is that when the intrathoracic pressure increases significantly, blood-borne emboli drift upward through the venous plexus to the venous sinuses of the skull (20). However, the specific mechanism needs to be further clarified.

In this study, we comprehensively analyzed various causes of clown nose-like lesions from our clinical cases and published literatures to further guide clinical diagnosis. The causes of clown nose-like lesions may be metastatic visceral tumors, genetic syndromes, and primary diseases involving the nasal tip. Comprehensive evaluation of clown nose-like lesions (e.g., occurring time of clown nose-like lesion, the number/color/symptom of nodules or papules, gender preference, family history, genetic background, and concomitant systemic involvements) in combination with other clinical information (e.g., histopathological findings) is emphasized to narrow the diagnosis (Table 5). In conclusion, the term of clown nose-like lesion should be expanded to consider genetic syndromes and primary diseases that affect the nasal tip, in addition to cutaneous metastasis.

**Table 5:** Identification points of clown nose-like lesions.

	**Metastatic visceral tumors**	**Genetic syndromes**	**Primary nasal diseases**
Occurring time of clown nose-like lesion	Over 50 years old generally	Young age, even at birth	N/A
The number of nasal nodules or papules	Often solitary	Often multiple^#^	Solitary or multiple^*^
The color of nodules or papules	Often red or inflammatory	Often fresh-colored	Often red or inflammatory
The symptom of clown nose-like lesion	Asymptomatic	Asymptomatic	often tender or pruritic^&^
Gender	N/A, male preference	Female preference	N/A
Family history	None	Often positive	None
Genetic background	None	Positive	None
Accompanying involvements	Visceral tumors	Multiple system involvement	None
Prognosis	Poor	Depending on the other system(s) involved	N/A

# *Except for some syndrome, e.g., Tricho-Rhino-Phalangeal Syndrome*.

* *For nasal special infection, e.g., nasal leishmaniasis, nasal nodules are multiple*.

& *There are individual differences in tender or pruritic sensations, and some clownlike nose can be asymptomatic*.

*“N/A,” data not available*.

## Data Availability Statement

The original contributions presented in the study are included in the article/supplementary material, further inquiries can be directed to the corresponding author/s.

## Ethics Statement

The studies involving human participants were reviewed and approved by Sichuan Academy of Medical Sciences & Sichuan Provincial People's Hospital. Written informed consent to participate in this study was provided by the participants' legal guardian/next of kin. Written informed consent was obtained from the individual(s), and minor(s)' legal guardian/next of kin, for the publication of any potentially identifiable images or data included in this article.

## Author Contributions

BZ contributed to literature search, data analysis, and drafting of the manuscript. LC contributed to conception of the study, case collection/analysis, literature search, and critical revision of the manuscript. JL and XL contributed to case collection/analysis. ZX contributed to case collection/analysis and constructive discussions. ZS contributed to conception and design of the study, literature search, data collection, and critical revision of the manuscript. All authors have approved the submitted version.

## Conflict of Interest

The authors declare that the research was conducted in the absence of any commercial or financial relationships that could be construed as a potential conflict of interest.

## References

[1] Colletti G Allevi F Moneghini L Palvarini M. Clown nose: a case of disfiguring nodular squamous cell carcinoma of the face. BMJ Case Rep. (2014) 2014:bcr2013200471. 10.1136/bcr-2013-200471 PMC3912395 24488659

[2] Chun SM Kim YC Lee JB Kim SJ Lee SC Won YH. Nasal tip cutaneous metastases secondary to lung carcinoma: three case reports and a review of the literature. Acta Derm Venereol. (2013) 93:569–72. 10.2340/00015555-1529 23303432

[3] Gomez-Zubiaur A Trasobares-Marugán L Aboín-González S Medina-Expósito I Villalobos-León ML. Solitary cutaneous metastasis of renal clear cell carcinoma on nasal tip. Melanoma Res. (2016) 26:E108–9. 10.1186/1477-7800-3-27

[4] Türegün M Bozkurt M Sengezer M Külahçi Y. Nasal tip metastasis of pharyngeal rhabdomyosarcoma. Ann Plast Surg. (2001). 46:656. 10.1097/00000637-200106000-00019 11405372

[5] Itin PH Heitzmann F Stamm B. Metastasis to the nasal tip from a cervical carcinoma. Dermatology. (1999). 199:171–3. 10.1159/000018231 10559590

[6] Knight TE Woo AS Jr. Blaisdell JM. Hepatocellular carcinoma invasive to chest wall. Int J Dermatol. (1992) 31:273–6. 10.1111/j.1365-4362.1992.tb03570.x 1321795

[7] Li L Mao GF Zhao XF Li X Wang YR Chang GY. A case of Tricho-Rhino-Phalangeal syndrome with new nonsense mutation and literature review. J Clin Pediatr. (2020) 38:306. 10.3969/j.issn.1000-3606.2020.04.015

[8] Santoro D Prisco M Ciaramella P. Cutaneous sterile granulomas/pyogranulomas, leishmaniasis and mycobacterial infections. J Small Anim Pract. (2008) 49:552–61. 10.1111/j.1748-5827.2008.00638.x 19006488

[9] Sand M Sand D Thrandorf C Paech V Altmeyer P Bechara FG. Cutaneous lesions of the nose. Head Face Med. (2010) 6:7. 10.1186/1746-160X-6-7 20525327 PMC2903548

[10] Netscher DT Wigoda P Green LK Spira M. Keratoacanthoma: when to observe and when to operate and the importance of accurate diagnosis. South Med J. (1994) 87:1272–6. 10.1097/00007611-199412000-00013 7973929

[11] Pichler M Thuile T Kluge R Puviani M Benedicenti F Eisendle K. Metastatic cylindrocarcinoma in Brooke-Spiegler Syndrome - report of a case and review of the literature. J Dtsch Dermatol Ges. (2021) 19:125–8. 10.1111/ddg.14227 32833298

[12] Gault DT Subbuswamy SG. Metastatic tumours of the nasal tip. Br J Plast Surg. (1985) 38:570–4. 10.1016/0007-1226(85)90023-2 2996670

[13] Nesi R Lynfield Y. Rhinophymalike metastatic carcinoma. Cutis. (1996) 57:33–6. 8620683

[14] Gal TJ Kerschner JE. Pulmonary metastasis to the nasal tip. Otolaryngol Head Neck Surg. (1997) 117:139. 10.1016/S0194-5998(97)70226-2 9230343

[15] De Simoni I Iacovelli P Lunghi F Peris K Chimenti S. “Clown nose” as a first manifestation of lung carcinoma. Acta Derm Venereol. (1997) 77:406–7. 9298146 10.2340/0001555577406407

[16] Vieira Mota A Correia O Resende C Azevedo F Mesquita-Guimarães J. Nasal tip metastasis revealing a Pancoast tumour. Br J Dermatol. (1998) 138:559–60. 10.1046/j.1365-2133.1998.02156.x 9580832

[17] Hammert WC Champagne L Heckler FR. Metastatic squamous cell carcinoma of the nasal tip. a case report. J Oral Maxillofac Surg. (1999) 57:186–9. 10.1016/S0278-2391(99)90237-9 9973129

[18] Rubinstein RY Baredes S Caputo J Galati L Schwartz RA. Cutaneous metastatic lung cancer: literature review and report of a tumor on the nose from a large cell undifferentiated carcinoma. Ear Nose Throat J. (2000) 79:96–7, 100–1. 10.1177/014556130007900209 10697933

[19] Camarasa Escrig A Chiner Vives E Sancho Chust JN. Clown nose as an initial manifestation of squamous-cell lung carcinoma. Arch Bronconeumol. (2009) 45:60–1. 10.1016/S1579-2129(09)71790-2 19186301

[20] Kataoka A Nishida T Tomioka Y Hirai N Ohbuchi M Yakushiji M. A metastasis to the nasal tip from a cervical carcinoma–a case report. Kurume Med J. (1998) 45:127–31. 10.2739/kurumemedj.45.127 9658762

[21] Ruiz HA Goldberg LH Humphreys TR Blacklock JB. Cutaneous metastasis of chordoma. Dermatol Surg. (2000) 26:259–62. 10.1046/j.1524-4725.2000.09216.x 10759805

[22] Brama I Goldfarb A Shalev O Ariel I. Tumour of the nose as a presenting feature of leukaemia. J Laryngol Otol. (1982) 96:83–7. 10.1017/S0022215100092252 6948901

[23] Shvili Y Talmi YP Gal R Kessler E Kolkov Z Zohar Y. Basaloid-squamous carcinoma of larynx metastatic to the skin of the nasal tip. J Craniomaxillofac Surg. (1990) 18:322–4. 10.1016/S1010-5182(05)80541-4 2262555

[24] Koutis EV Assimakopoulos DA Doukas MG Zinovieva I. A rare nasal tip skin metastasis of a basaloid squamous cell carcinoma of the larynx. Am J Med. (2008) 121:e3–4. 10.1016/j.amjmed.2008.04.029 18724953

[25] Kohli PS Soni NK. Nasal tip metastasis. an unusual site and mode of spread in anaplastic thyroid carcinoma. Indian J Otolaryngol Head Neck Surg. (2008) 60:269–70. 10.1007/s12070-008-0039-4 23120560 PMC3450640

[26] Shindo M Yoshida Y Tominaga K Yamamoto O. Skin metastasis of hypopharyngeal carcinoma to the nasal tip. Yonago Acta Med. (2013) 56:57–8. 24031153 PMC3771206

[27] Kocak Z Uygun K Uzal MC Cicin I Yalcin O. Unusual metastatic site in a case of carcinoma of the hypopharynx: nasal tip. J Otolaryngol. (2005) 34:250–2. 10.2310/7070.2005.34409 16048695

[28] Dong A Zuo C Wang Y Zhai Z Wen W. Isolated nasal tip metastasis from esophageal squamous cell carcinoma. Clin Nucl Med. (2015) 40:65–7. 10.1097/RLU.0000000000000387 24566414

[29] Chau CH Siu WT Li MK. Nasal tip metastasis from esophageal carcinoma. Can J Surg. (2002) 45:224–5. 12067183 PMC3686961

[30] Ledderose GJ Englhard AS. Isolated nasal tip metastasis from esophageal squamous cell carcinoma: case report and literature review. Case Rep Otolaryngol. (2015). 2015:246094. 10.1155/2015/246094 26175919 PMC4484564

[31] Melo DG Acosta AX de Pina-Neto JM. Syndrome of psychomotor retardation, bulbous nose, and epilepsy (Hernandez syndrome): a Brazilian case. Clin Dysmorphol. (1999) 8:301–3. 10.1097/00019605-199910000-00015 10532183

[32] Zhao XY Huang YJ Liang YH Huang L Zhao Y Zeng K. Multiple familial trichoepithelioma. report of a Chinese family not associated with a mutation in the CYLD gene and CYLD protein expression in the trichoepithelioma tumor tissue. Int J Dermatol. (2014) 53:e279–81. 10.1111/ijd.12153 23879700

[33] Sayed CJ Matheis P Morrell DS. Hypotrichosis, bulbous nose, and cone-shaped epiphyses in an 8-year-old girl. Trichorhinophalangeal syndrome type I. Pediatr Dermatol. (2008) 25:557–8. 10.1111/j.1525-1470.2008.00760.x 18950401

[34] Trippella G Lionetti P Naldini S Peluso F Monica MD Stagi S. An early diagnosis of trichorhinophalangeal syndrome type 1. a case report and a review of literature. Ital J Pediatr. (2018) 44:138. 10.1186/s13052-018-0580-z 30458885 PMC6245908

[35] Falsafi P Taghavi-Zenouz A Khorshidi-Khiyavi R Nezami N Estiar MA. A case of tuberous sclerosis without multiorgan involvement. Glob J Health Sci. (2015) 7:124–31. 10.5539/gjhs.v7n5p124 26156917 PMC4803869

[36] Nakano H Otsuka A Kinoshita M. A subtle case of tuberous sclerosis complex. Epilepsy Behav Case Rep. (2015) 4:88–90. 10.1016/j.ebcr.2015.08.002 26543814 PMC4589839

[37] Salman A Yucelten AD Seckin D Ergun T Demircay Z. Cutaneous leishmaniasis mimicking verrucous carcinoma: a case with an unusual clinical course. Indian J Dermatol Venereol Leprol. (2015) 81:392–4. 10.4103/0378-6323.157462 25994897

[38] Abdalla M Sax PE Mostaghimi A Miller AL Loscalzo J. Clinical problem-solving. On the nose. N Engl J Med. (2015) 373:955–61. 10.1056/NEJMcps1315433 26332551

[39] Vu A Laub D Jr. Metastatic Basal cell carcinoma: a case report and review of the literature. Eplasty. (2011) 11:ic8. 10.5772/26604 21559224 PMC3088016

[40] Osada S Ueno T Inai S Niimi Y Nakamizo M Ansai S. Sebaceous carcinoma of the nose with a regional metastasis following false-negative sentinel lymph node biopsy. Acta Derm Venereol. (2011) 91:367–8. 10.2340/00015555-1047 21461553

[41] Ravaioli GM Lambertini M Pazzaglia M Corti B Fanti PA Dika E. Pilomatrix carcinoma of the nose: clinical and dermoscopic presentation. JAAD Case Rep. (2018) 4:376–8. 10.1016/j.jdcr.2017.12.001 29693075 PMC5911817

[42] Bewer F Förster C Welkoborsky HJ. Microcystic adnexal carcinoma (malignant syringoma) of the nose: case report and review of the literature. Laryngorhinootologie. (2004) 83:113–6. 10.1055/s-2004-814107 14999587

[43] Miyamoto T Yoshino T Takehisa T Hagari Y Mihara M. Cutaneous presentation of nasal/nasal type T/NK cell lymphoma: clinicopathological findings of four cases. Br J Dermatol. (1998) 139:481–7. 10.1046/j.1365-2133.1998.02414.x 9767295

[44] Wong TY Suster S. Primary cutaneous sarcomas showing rhabdomyoblastic differentiation. Histopathology. (1995) 26:25–32. 10.1111/j.1365-2559.1995.tb00616.x 7713481

[45] Suarez-Peñaranda JM Vieites B Ramírez-Santos A Fernández-Redondo V Toribio J Del Rio E. Clinicopathological and immnuohistochemical findings in a series of folliculosebaceous cystic hamartoma. J Cutan Pathol. (2009) 36:251–6. 10.1111/j.1600-0560.2008.01011.x 18715254

[46] Ribeiro BNdF Marchiori E. Rosai-Dorfman disease affecting the nasal cavities and paranasal sinuses. Radiol Bras. (2016) 49:275–6. 10.1590/0100-3984.2015.0167 27777489 PMC5073402

[47] Mallis A Mastronikolis NS Koumoundourou D Stathas T Papadas TA. Sinonasal sarcoidosis. A case report. Eur Rev Med Pharmacol Sci. (2010) 14:1097–9. 21375142

[48] Aburezq H Jaeger M Iyengar P Zuker R. Juvenile xanthogranuloma of the nose. Can J Plast Surg. (2004) 12:198–200. 10.1177/229255030401200406 24115896 PMC3792822

[49] Sittart JAdS Senise M. Cutaneous metastasis from internal carcinomas: a review of 45 years. An Bras Dermatol. (2013) 88:541–4. 10.1590/abd1806-4841.20131165 24068124 PMC3760928

[50] Fares J Fares MY Khachfe HH Salhab HA Fares Y. Molecular principles of metastasis. a hallmark of cancer revisited. Signal Transduc Target Ther. (2020) 5:28. 10.1038/s41392-020-0134-x 32296047 PMC7067809

[51] Popper HH. Progression and metastasis of lung cancer. Cancer Metastasis Rev. (2016) 35:75–91. 10.1007/s10555-016-9618-0 27018053 PMC4821869

[52] Bain C Feskanich D Speizer FE Thun M Hertzmark E Rosner BA. Lung cancer rates in men and women with comparable histories of smoking. J Natl Cancer Inst. (2004) 96:826–34. 10.1093/jnci/djh143 15173266

[53] García M. Clown nose as a metastatic manifestation in skin of a lung cancer. Archivo Médico de Camagüey. (2018) 22:531–9.

[54] Itin PH Bohn S Mathys D Guggenheim R Richard G. Trichorhinophalangeal syndrome type III. Dermatology. (1996) 193:349–52. 10.1159/000246290 8993967

[55] Spencer PS Helm TN. Skin metastases in cancer patients. Cutis. (1987) 39:119–21. 3829718

[56] Rosen T. Cutaneous metastases. Med Clin North Am. (1980) 64:885–900. 10.1016/S0025-7125(16)31572-3 7432046

[57] Lookingbill DP Spangler N Sexton FM. Skin involvement as the presenting sign of internal carcinoma. A retrospective study of 7316 cancer patients. J Am Acad Dermatol. (1990) 22:19–26. 10.1016/0190-9622(90)70002-Y 2298962

[58] Batson OV. The function of the vertebral veins and their role in the spread of metastases. Ann Surg. (1940) 112:138–49. 10.1097/00000658-194007000-00016 17857618 PMC1387927

[59] Ruggeri CS Acosta L Proietti V Serrano C. Nasal cavity and paranasal sinuses metastasis. Int J Cancer Clin Res. (2020) 7:143. 10.23937/2378-3419/141014319560300

